# Loss of Hypoxia Signaling Limits Physiologic and Muscle Adaptations to Aerobic Exercise in Aging

**DOI:** 10.1093/geroni/igab046.2554

**Published:** 2021-12-17

**Authors:** Yori Endo, Mehran Karvar, Yuteng Zhang, Shayan Olumi, Indranil Sinha

**Affiliations:** 1 Harvard Medical School, Boston, Massachusetts, United States; 2 Brigham and Women's Hospital, Harvard Medical School, Boston, Massachusetts, United States; 3 Northwestern University, Evanston, Illinois, United States; 4 Birgham and Women's Hospital, Harvard Medical School, Boston, Massachusetts, United States

## Abstract

To assess the differential effects of exercise with age, Young (Y, 10-12 weeks) and Old (O, 23-25 months) mice were subjected to regimented treadmill running or no regimented exercise. Y, trained mice experienced a significant increase in maximal distance running, maximal speed of running, and lean muscle mass in comparison to age-matched, untrained controls. O mice did not improve significantly in any of these measures following training. Transcriptome analysis of gastrocnemius from Y mice demonstrated differential regulation of 120 genes with exercise. None of these genes were similarly regulated in the O group. Genes most upregulated following exercise in Y mice were direct targets of the hypoxia signaling pathway. Immunoblotting demonstrated that aryl hydrocarbon receptor nuclear translocator (ARNT), a critical regulator of hypoxia signaling, increased 3-fold with exercise in Y mice, but this increase was absent in O mice following exercise. To assess whether this loss of ARNT in O muscle impaired the exercise response, we generated a mouse with inducible, skeletal muscle-specific knockout of ARNT (ARNT muscle (m) KO). Following regimented exercise, ARNT mKO mice did not improve maximal distance running, maximal running speed, or lean muscle mass in comparison to untrained ARNT mKO mice. Littermate, age-matched ARNT wild type mice increased significantly in all of these measures following training. Administration of ML228, an ARNT agonist, increased maximal running distance and speed in response to exercise training in O mice. These results suggest that restoration of ARNT and hypoxia signaling may restore the physiologic response to exercise in aging.

